# Prevalence and identification of caprine pasteurellosis in pneumonic goats in Bangladesh

**DOI:** 10.5455/javar.2023.j707

**Published:** 2023-09-30

**Authors:** Md. Habibur Rahman, Sonia Akther, Md. Shahin Alam, Md. Zakir Hassan, Md. Samun Sarker, Md. Zulfekar Ali, Md. Giasuddin, Sadek Ahmed

**Affiliations:** 1 Goat Production Research Division, Bangladesh Livestock Research Institute, Savar, Dhaka-1341, Bangladesh; 2 Sheep Production Research Division, Bangladesh Livestock Research Institute, Savar, Dhaka-1341, Bangladesh; 3 Animal Health Research Division, Bangladesh Livestock Research Institute, Savar, Dhaka-1341, Bangladesh

**Keywords:** Bangladesh, Goat, *Mannheimia haemolytica*, *Pasteurella multocida*, pasteurellosis

## Abstract

**Objective:**

This research aimed to assess the prevalence of caprine pasteurellosis, isolate and identify pasteurellosis (*Mannheimia haemolytica* and *Pasteurella multocida*) in pneumonic goats, and discover the main bacterial cause of pneumonia.

**Materials and Methods:**

One hundred and five samples (94 nasal swabs and 11 lung tissues) from goats suspected of having pneumonia were taken and transferred aseptically to the laboratory. Following the processing of the collected samples, *Pasteurella* spp. was isolated with the aid of plate culture methods. Biochemical characteristics were used to identify all bacterial isolates, which were then verified by polymerase chain reaction (PCR). Antimicrobial susceptibility testing was also carried out to evaluate the sensitivity profiles of various antibiotics. The *Pasteurella haemolytica* serotype-specific antigen (PHSSA) gene was used to identify isolates of *M. haemolytica*, and the KMT1 gene was used to identify isolates of *P. multocida*.

**Results:**

From the 105 clinically suspicious samples, 51 (48.57%) were identified to be *Pasteurella spp.* through bacteriological testing and also by PCR targeting the *16S rRNA* gene. Of these, 47.87% (45/94) were nasal swabs, and 54.55% (6/11) were lung tissues. Among confirmed samples, 70.59% (36/51) were identified as *M. haemolytica,* and 29.41% (15/51) were identified as *P. multocida*. Resistance to tetracycline, streptomycin, oxytetracycline, gentamicin, and ceftriaxone was found in 50%–83% of the isolates. In addition, PCR identified the PHSSA and KMT1 genes from isolates of *P. multocida* and *M. haemolytica*, respectively.

**Conclusion:**

The present study revealed that *M. haemolytica* and *P. multocida* primarily caused pasteurellosis in pneumonic goats in Bangladesh. However, when treating these animals, the proper choice of antimicrobials should be made to control this disease.

## Introduction

The goat is called the poor man’s cow in Bangladesh and is the second-most important livestock species. Currently, there are about 26.8 million goats in Bangladesh, and the gross domestic product (GDP) contribution of livestock is 1.90%, while the GDP growth rate of livestock is 3.10% in the National Economy of Bangladesh in 2021–22 [1]. Various diseases cause huge morbidity and mortality in goats, thus causing productivity losses, ultimately affecting the economy of goat farming, and increasing the veterinary cost [2,3]. Goats suffer from different diseases, and a portion of these are typical of other domesticated animal species. At the same time, some diseases are intended for goats only, and some are zoonotic [4].

Pneumonia is one of the major problems for goats not only in Bangladesh but also throughout the world. One of the main issues with goats worldwide, not just in Bangladesh, is pneumonia. According to Marru et al. [5], it is one of the most prevalent respiratory diseases in goats worldwide. According to Jesse Abdullah et al. [6], anorexia, fever (40°C–41°C), persistent cough, breathlessness, mucopurulent discharge from the nose, and lethargy are all symptoms of pneumonia in goats. The most prevalent respiratory tract infection in ruminant animals, pneumonic pasteurellosis, generates losses due to high mortality, expensive treatment, poor weight increase, delayed marketing, and unfrugality among flock survivors [7]. Goats and sheep are susceptible to the disease, which they catch when stressed or in an unfavorable environment [8]. The two bacteria *Mannheimia haemolytica* and *Pasteurella multocida* are the most common causes of bacterial pneumonia. They are more frequently linked to outbreaks of acute pneumonia and goat deaths across all age categories [9,10].

One of the most significant respiratory infections in domestic ruminants, *P. multocida,* is an opportunistic bacterium similar to *M. haemolytica* [11]; it produces severe epidemics of acute pneumonia [5,6]. When stressors like weaning stress and long shipping, environmental change, or infections brought on by multiple primary respiratory pathogens weaken an animal‘s immune system, a respiratory environment that is conducive to the colonization and replication of several pathogenic bacteria is created [12,13]. These two bacteria are common and normal nasopharyngeal commensals in animals in good health, and they only become harmful when their hosts‘ natural defenses are exhausted [13,14].

Pneumonic pasteurellosis affects goats in Bangladesh, but there is a lack of published information on the disease. In Bangladesh, pneumonia has recently had a serious impact on peste des petits ruminants (PPR)-vaccinated goats. Pasteurellosis is a PPR-like disease, so farmers are normally confused with PPR. So, this study was carried out to determine the prevalence of caprine pasteurellosis, isolate and identify pasteurellosis (*M. haemolytica* and *P. multocida*) in pneumonic goats, and discover the main bacterial cause of pneumonia in PPR-vaccinated goats in Bangladesh. The current study not only helped to provide some precise information about caprine pasteurellosis and its causal agent but also helped to take necessary remedial action for sustainable goat production in Bangladesh.

## Materials and Method

### Ethical approval 

The Animal Experimentation Ethics Committee of the Bangladesh Livestock Research Institute approved this experiment (Reference No.: AEEC/BLRI00110/2023). All the rules and regulations for animal care were strictly maintained during sample collection.

### Study area and population

A total of 105 samples (94 nasal swabs and 11 lung tissues) have been collected from goats suspected of having pneumonia from different parts of Bangladesh, including Savar (nasal-13, lung-6), Bhaluka (nasal-12, lung-0), Muktagacha (nasal-9, lung-0), Jashore (nasal-16, lung-0), Chuadanga (nasal-11, lung-0), Meherpur (nasal-13, lung-0), Kustia (nasal-8, lung-0), and Rajshahi (nasal-12, lung-5). These goats became physically sick and displayed anorexia, mucopurulent discharge from the nose, severe cough and fever.

### Rearing system and vaccination history

All the animals were PPR-vaccinated and reared in both a free-ranging and a semi-intensive system.

### Collection and processing of samples

All the ill animals exhibiting respiratory symptoms had their nasal swabs (*n = *94) collected into 2 ml of brain heart infusion (BHI) broth (Oxoid, UK). Animals that had died were necropsied, and lung tissue samples (*n = *11) were taken. The outer surface of the pneumonic lungs was cleaned with a heated spatula before cutting its interior part for sampling in the postmortem examination. Each animal‘s inner lung tissue was removed using aseptic techniques to separate *P. multocida* and *M. haemolytica*. The lung tissue samples were treated in an aseptic manner by creating a tissue homogenate with a weight-to-volume ratio of 20% of phosphate buffer saline inside a laminar airflow cabinet close to a flame.

### Bacterial isolation and identification

Each sample has been streaked directly into a blood agar base (Oxoid, UK) enriched with 7% defibrinated goat blood and incubated under aerobic conditions at 37°C for 24–48 h. After that, the colonies‘ morphology, color, and odor were studied, along with the plates‘ bacterial growth. Gram staining was used on the suspicious colonies, and they were inspected and examined for the following traits: hemolysis on blood agar, motility, production of oxidase and indole, and growth on MacConkey agar (Oxoid, UK). To further identify *P. multocida* or *M. haemolytica*, the identification of Gram-negative coccobacilli was done by the above-mentioned conventional culture, and biochemical assays were used [15]. For later usage, the pure bacterial colonies were kept in BHI agar (Oxoid, UK) slants.

### Antimicrobial susceptibility testing

The susceptibility of all *P. multocida* and *M. haemolytica* isolates to gentamicin (10 μg), tetracycline (30 μg), ceftriaxone (30 μg), streptomycin (10 μg), and oxytetracycline (30 μg) disks (Oxoid, UK) was assessed through the disc diffusion method [16]. The quality control strain was *Escherichia coli* ATCC 25922. The Clinical and Laboratory Standards Institute breakpoints [17] were used to interpret the results.

### Isolation of deoxyribonucleic acid (DNA) from bacterial colonies

To isolate the genomic DNA, the presumed *M. haemolytica* and *P. multocida* isolates were streaked on BHI agar (Oxoid, UK). This was done aerobically at 37°C for 48 h. The pure isolates of *P. multocida* and *M. haemolytica* were put into 2 ml microcentrifuge tubes with around 4–5 colonies of each. Using a micro-centrifuge, the bacterial colonies were centrifuged two times with nuclease-free water for 3 min. According to the manufacturer‘s recommendations, the genomic DNA was extracted using the DNeasy blood and tissue kit (Qiagen, USA).

### Polymerase chain reaction (PCR) amplification

The bacterial isolates were identified by PCR amplification using the specified primers (Macrogen, South Korea) (Table 1) [18–20]. All of the genes had their reaction mixes and amplification conditions optimized. The 16S rRNA gene amplification was done with universal primers, and isolates of *M. haemolytica* and *P. multocida* were particularly detected. Additionally, species-specific amplification of the *Pasteurella haemolytica* serotype-specific antigen gene (PHSSA) and KMT1 genes, respectively, allowed for the identification of *M. haemolytica* and *P. multocida*. A 25 μl reaction volume, including deionized water (10.5 μl), GoTaq master mix (Promega, USA) (12.5 μl), forward and reverse primers (0.5 μl each), and DNA template (1 μl) was used to perform PCR amplification. For the PHSSA PCR, the following conditions were used: a 3 min initial denaturation at 95°C, 35 cycles of 1 min denaturation at 95°C, 1 min annealing at 48°C, 30 sec extension at 72°C, and 5 min of final extension at 72°C. The KMT1 gene was subjected to PCR settings that included an initial denaturation at 95°C for 3 min, 35 cycles that included denaturation at 95°C for 45 sec, annealing at 56°C for 45 sec, extension at 72°C for 1 min, and the final extension at 72°C for 5 min. The amplified PCR products (5 μl) were separated in 1.5% *w*/*v* agarose gel and stained with ethidium bromide using horizontal submarine electrophoresis equipment. The gel documentation system was used to examine the results.

## Results

Gram-negative coccobacilli and tiny bacilli, suspected to be *P. multocida* and/or *M. haemolytica* were isolated (*n = *51) using bacterial culture. A classical identification of the isolates was made using cultural and biochemical traits. On the blood agar, small, slick, white-creamy, mucoid, hemolytic, and nonhemolytic colonies emerged. After 48 h, the MacConkey agar was further streaked with hemolytic colonies, which thereafter displayed modest growth. The MacConkey agar did not support the growth of the nonhemolytic colonies. On deoxycholate citrate agar (Oxoid, UK), none of the isolates displayed any growth. Gram staining of *P. multocida* putative isolates revealed tiny Gram-negative coccobacilli. The colonies that were thought to be *M. haemolytica*, however, had pleomorphic, Gram-negative, coccobacillus-to-small bacillary morphology. Catalase and oxidase production were both present in all of the samples. *Pasteurella multocida* isolates yielded indole.

**Figure 1.**
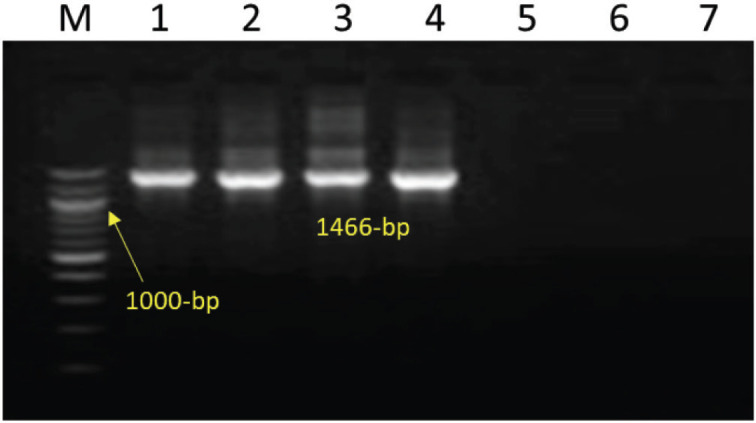
Amplification of 16S rRNA gene (1466-bp) of *Pasteurella *spp*.* Lanes 1-4: test positive. Lanes 5, 6, 7: test negative. Lane M: 100-bp DNA ladder.

**Figure 2.**
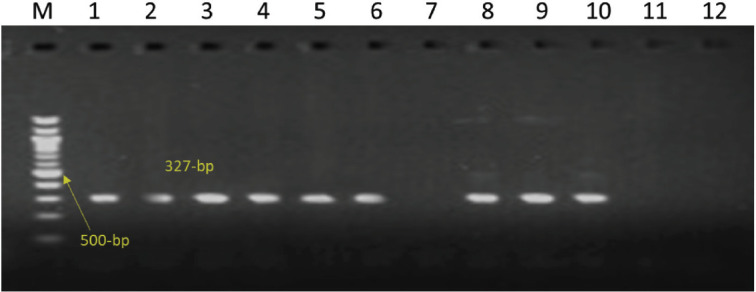
Amplification of PHSSA gene (327-bp) of *M. haemolytica*. Lanes 1–6 and Lanes 8–10: test positive. Lanes 7, 11, 12: test negative. Lane M: 100-bp DNA ladder.

**Figure 3.**
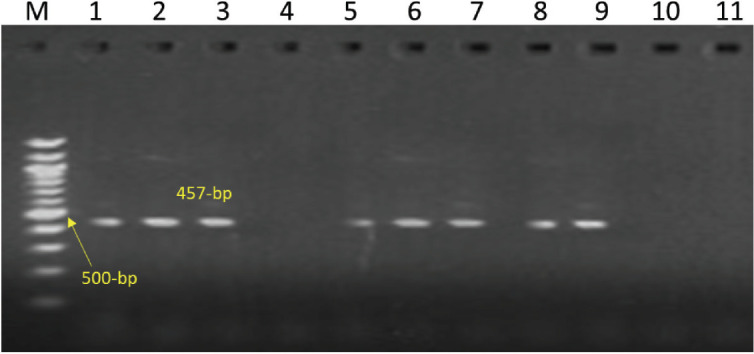
Amplification of KMT1 gene (457-bp) specific to *P. multocida*. Lanes 1-3 and Lanes 5-9: test positive. Lanes 4, 10, 11: test negative. Lane M: 100-bp DNA ladder.

The 16S rRNA gene amplification with universal primers is shown in Figure 1. By specifically amplifying the PHSSA (327 bp) and KMT1 genes (457 bp), respectively, isolates of *M. haemolytica* and *P. multocida *were further identified, as shown in Figures 2 and 3.

**Table 1.** List of primers used in PCR.

Gene	Primer sequence	Amplicon size (bp)	Annealing temp (°C)	References
16S rRNA	F 5′-AGAGTTTGATCMTGGCTCAG-3′ R 5′-CGGTTACCTTGTTACGACTT-3′	~1,466	52	[18]
PHSSA	F 5′-TTCACATCTTCATCCTC-3′ R 5′-TTTTCATCCTCTTCGTC-3′	327	48	[19]
KMT1	F 5′-ATCCGCTATTTACCCAGTGG-3′ R 5′-GCTGTAAACGAACTCGCCAC-3′	457	56	[20]

From the 105 clinically suspected samples, 51 (48.57%) were confirmed as *Pasteurella *spp. by PCR, 47.87% (45/94) were nasal swabs, and 54.55% (6/11) were lung tissue, as shown in Table 2. Among confirmed isolates, 70.59% (36/51) were *M. haemolytica* and 29.41% (15/51) were *P. multocida* as shown in Table 3.

### Antimicrobial susceptibility testing

**Figure 4.**
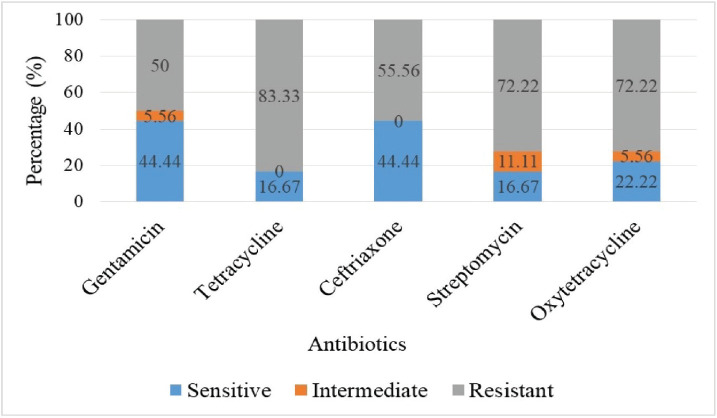
Antibiotic susceptibility results of *Pasteurella* spp.

The highest resistance was observed to tetracycline (83.33%), streptomycin (72.22%), and oxytetracycline (72.22%). All of the tested isolates, including gentamicin (44.44%) and ceftriaxone (44.44%), were the most sensitive antibiotics (Figure 4).

## Discussion

In Bangladesh, a variety of rural families rely on goat farming to make a living. However, the goats are more susceptible to developing pneumonia due to fluctuating environmental variables. Globally, sheep and goat populations are affected by pneumonic pasteurellosis, which might be acute in nature and cause sudden mortality, breathing difficulties, drowsiness, anorexia, and fever [21]. Physical environmental pressures weaken the goats‘ natural resistance to *P. haemolytica* infection, which makes them more susceptible to respiratory infections [8]. According to Rahal et al. [22] and Galapero et al. [23], abrupt environmental changes, including wind, temperature, and rainfall, have been directly linked to the prevalence and progression of pneumonia among sheep and goats. These animals are more susceptible to pasteurellosis due to significant elements, including production stress, environmental conditions in a specific location, and shifting weather patterns [7]. Pasteurellosis has been identified as the primary small ruminant disease associated with climate change, according to a study conducted by 126 World Organization for Animal Health member countries [24]. In rural settings, it is crucial to identify infections such as pneumonia in livestock using necropsy-based gross pathology observations. For the National Disease Reporting System and record-keeping, such an infectious disease diagnosis is essential. According to a necropsy, fibrinous bronchopneumonia with lung and tracheal hemorrhages and congestion was observed comparable to past investigations [25]. The apical lobe of pneumonic lungs showed consolidation [26]. The necropsy of lung lobes from goats that died of pneumonia pasteurellosis revealed hemorrhagic (bloody) secretions, along with possible pus and dead tissue lesions.

**Table 2.** Prevalence of *Pasteurella *spp. from different types of samples.

Type of sample	Number of samples	PCR positive (%)	95% Confidence interval (CI)
Nasal samples	94	45(47.87)	38.06–57.85
Lung samples	11	6(54.55)	28.01–78.73
Total	105	51(48.57)	39.23–58.01

**Table 3.** Species wise prevalence by PCR (*P. multocida *and *M. haemolytica*).

Organisms	Number of samples tested	PCR positive (%)	95% CI
*Pasteurella *spp.	105	51 (48.57%)	39.23–58.01
*Pasteurella multocida *	51	15 (29.41%)	18.71–43
*Mannheimia haemolytica*	51	36 (70.59%)	57–81.29

It is important to note that *P. multocida* and *M. haemolytica* usually appear as typical elements of the nasal and pharyngeal microbiota of healthy animals. Although, according to Mohamed and Abdelsalam [8], detection of this bacteria in the lower respiratory system typically denotes the presence of a clinical state. In his review, Ugochukwu [27] noted that various researchers had also conducted experiments that demonstrated how this particular organism could flare up on its own or along with other pathogens to produce serious infections that have a significant death and morbidity rate under specific circumstances related to debilitation, nutrition, and climatic factors. According to Dassanayake et al. [28], respiratory syncytial virus, mycoplasma infection, and parainfluenza-3 virus infections are the main causes of pneumonia in small ruminants. Variable climatic conditions further increase the risk of pneumonia. Infections in the lower respiratory system due to respiratory viruses alter the lungs‘ mucociliary clearance systems, which make small ruminants more vulnerable to subsequent infections caused by bacteria [9]. However, pneumonic pasteurellosis and respiratory mannheimiosis are thought to be mostly caused by *M. haemolytica*, one of the common bacteria identified in lung samples [29]. Parainfluenza-3 virus and the respiratory syncytial virus may lead to nonfatal pneumonia, but they are not necessarily risk factors for *M. haemolytica*-induced pneumonia in bighorn sheep [29]. The investigation of viruses and mycoplasma was not conducted for this study. According to Dabo et al. [30], *M. haemolytica* greatly improves *P. multocida* colonization, which results in a more serious illness.

Tetracycline and oxytetracycline resistance were present in the isolates, probably as a result of the flock‘s excessive usage of these antibiotics. The current study‘s susceptibility findings were more or less consistent with those of the other investigations [31,32]. It is possible that the flock‘s selective usage of beta-lactams (ceftriaxone) and aminoglycosides (gentamicin) contributed to their reduced susceptibility. The aggressive *M. haemolytica* and/or *P. multocida* strains demonstrated their involvement in the progression of goat pneumonia and the evolution of this disease. According to Tamil Nadu, India‘s *P. multocida* Type A participation in sheep encountered with pneumonia has been documented [33], supporting the results of the present investigation. Similar to the current investigation, previous reports have described *M*.* haemolytica* and *P. mul*tocida isolation and identification on the basis of culture method and biochemical features, as well as PCR tests addressing the particular genes. In addition, according to Singh et al. [34], Singh et al. [35] and Hassan et al. [36], the exact molecular detection of *P. multocida* using 16S rRNA and the KMT1 gene has a substantial effect on the epidemiology and management of pasteurellosis in small ruminants. In addition, it is asserted that molecular typing techniques enable quick identification of bacteria and have been shown to be more accurate than culture and biochemical testing [,.

## Conclusion

Pneumonic pasteurellosis is one of the PPR-like diseases that affect goats most frequently. This study reveals that caprine pasteurellosis in pneumonic goats is primarily caused by *M. haemolytica* followed by *P. multocida* in Bangladesh, which considerably damages goat populations and results in significant losses by increasing morbidity and mortality rates. Further molecular studies are recommended to uncover the root cause analysis of caprine pasteurellosis in goats.

## List of Abbreviations

BHI agar, brain heart infusion agar; DNA, deoxyribonucleic acid; GDP, gross domestic product; *M. haemolytica,* *Mannheimia haemolytica*; PCR, polymerase chain reaction; PHSSA gene, *Pasteurella haemolytica* serotype-specific antigen gene; *P. multocida, Pasteurella multocida*; PPR, Peste des Petits ruminants; UK, United Kingdom; USA, United States of America; μg, microgram.
